# Metabolite Measurement in Index Substrate Drug Interaction Studies: A Review of the Literature and Recent New Drug Application Reviews

**DOI:** 10.3390/metabo14100522

**Published:** 2024-09-26

**Authors:** Jingjing Yu, Nathalie Rioux, Iain Gardner, Katie Owens, Isabelle Ragueneau-Majlessi

**Affiliations:** 1 Center of Excellence in Drug Interaction Science, Certara USA, 4 Radnor Corporate Center, Suite 350, Radnor, PA 19087, USA; nathalie.rioux@certara.com (N.R.); isabelle.ragueneau-majlessi@certara.com (I.R.-M.); 2 Center of Excellence in Drug Interaction Science, Certara UK, 1 Concourse Way, Sheffield S1 2BJ, UK; iain.gardner@certara.com; 3 Drug Interaction Solutions, Certara USA, 4 Radnor Corporate Center, Suite 350, Radnor, PA 19087, USA; katie.owens@certara.com

**Keywords:** drug interaction, metabolism, metabolite, pharmacokinetics

## Abstract

Background/Objectives: Index substrates are used to understand the processes involved in pharmacokinetic (PK) drug–drug interactions (DDIs). The aim of this analysis is to review metabolite measurement in clinical DDI studies, focusing on index substrates for cytochrome P450 (CYP) enzymes, including CYP1A2 (caffeine), CYP2B6 (bupropion), CYP2C8 (repaglinide), CYP2C9 ((S)-warfarin, flurbiprofen), CYP2C19 (omeprazole), CYP2D6 (desipramine, dextromethorphan, nebivolol), and CYP3A (midazolam, triazolam). Methods: All data used in this evaluation were obtained from the Certara Drug Interaction Database. Clinical index substrate DDI studies with PK data for at least one metabolite, available from literature and recent new drug application reviews, were reviewed. Further, for positive DDI studies, a correlation analysis was performed between changes in plasma exposure of index substrates and their marker metabolites. Results: A total of 3261 individual index DDI studies were available, with 45% measuring at least one metabolite. The occurrence of metabolite measurement in clinical DDI studies varied widely between index substrates and enzymes. Discussion and Conclusions: For substrates such as caffeine, bupropion, omeprazole, and dextromethorphan, the use of the metabolite/parent area under the curve ratio can provide greater sensitivity to DDI or reduce intrasubject variability. In some cases (e.g., omeprazole, repaglinide), the inclusion of metabolite measurement can provide mechanistic insights to understand complex interactions.

## 1. Introduction

Understanding the various processes involved in pharmacokinetic (PK) drug–drug interactions (DDIs) is critical to facilitate the optimal management of these DDIs in the clinic. Mechanistic methodologies have been used by the pharmaceutical industry to assess DDI risk during drug development. One aspect of these methodologies includes the evaluation of the potential of the investigational drug to affect the metabolism of other drugs by assessing its impact on the PK of recommended index substrates for cytochrome P450 (CYP) enzymes. Examples of index substrates for CYP enzyme have been recommended in various DDI regulatory guidances and have been harmonized in the recently released ICH M12 guidance [1]. For DDI studies, in general, metabolite concentrations should be determined “if they provide information about the effect of a DDI on safety or efficacy or if the data inform the mechanism of the drug interaction. For example, when a clinical DDI study evaluates drugs that may interact via multiple pathways, measuring metabolites may help determine the enzymes and/or transporters responsible for the interaction” [1]. Specifically, when an investigational drug is evaluated as an inhibitor or inducer of CYP enzymes and if the substrate drug is metabolized by more than one enzyme, measuring metabolites may help with the interpretation of study results [1].

Previous research has highlighted the importance of measuring metabolites of precipitant drugs, as circulating metabolites of precipitant drugs may play a significant role in in vivo inhibition of CYP enzymes [2]. Complex DDIs can arise from the inhibition or induction of these enzymes by major drug metabolites [3]. On the other hand, in the case of a CYP index drug, i.e., a drug that is almost entirely cleared by one enzyme, measuring metabolites may not be in theory as useful or necessary. A recent exploratory analysis of in-house DDI studies involving eight investigational drugs as precipitants and oral midazolam was conducted to assess the utility of measuring 1′-hydroxymidazolam in plasma. The analysis concluded that measuring 1′-hydroxymidazolam may not be valuable for interpreting results in CYP3A DDI studies for an investigational drug as a precipitant [4]. However, this conclusion does not extend to other index substrates or larger datasets, as no such analyses have been performed. The aim of this review was therefore to review the literature and Food and Drug Administration new drug application (NDA) reviews of recently approved drugs to evaluate metabolite PK measurement of the recommended index substrates included in clinical DDI studies.

## 2. Methods

All the data used in this analysis were obtained from the Certara Drug Interaction Database (DIDB; www.druginteractionsolutions.org), accessed on 13 May 2024. Clinical DDI studies with ICH recommended index substrates as the object for CYP1A2 (caffeine), CYP2B6 (bupropion), CYP2C8 (repaglinide), CYP2C9 ((S)-warfarin, flurbiprofen), CYP2C19 (omeprazole), CYP2D6 (desipramine, dextromethorphan, nebivolol), and CYP3A (midazolam, triazolam) with at least one metabolite measured (regardless of whether the measurement was in plasma, urine, or other biofluid), were extracted from the DIDB and further analyzed [1]. Tolbutamide was not recommended as an index substrate for CYP2C9 at the time of the data search and was therefore not included in this analysis. Data obtained from citations including both literature and recent NDA reviews were thoroughly reviewed. This dataset is referred to as index DDI studies in this analysis. Case reports and simulated DDI results from physiologically based pharmacokinetic (PBPK) and population PK studies were excluded. In general, a DDI study is considered positive if the area under the curve (AUC) change in the object drug is ≥1.25-fold for inhibition or ≤0.8-fold for induction. Additionally, a DDI study was also considered positive by the authors based on changes in object drug clearance or metabolite AUC. This dataset was further analyzed chronologically for data obtained from the literature (up to the end of 2023) and NDA reviews in the past 10 years (2014–2023), respectively. For the chronological analysis, 2024 data was not included as only part of the year was available. In addition, the numbers of all DDI studies and DDI studies with metabolite measured curated in the DIDB were mined.

For positive studies (those demonstrating substrate AUC changes ≥ 1.25-fold for inhibition or ≤0.8-fold for induction) where plasma measurements of both substrates and metabolites were reported, an exploratory correlation analysis was performed between changes in plasma exposure of index substrates and their metabolites. Specifically, changes in plasma AUC of parent index substrates, plasma AUC of marker metabolites, and metabolite/parent (M/P) AUC ratios (i.e., metabolic ratio) were compared. For substrates with multiple metabolites measured, only the primary metabolite mediated by the specific CYP, referred to as the marker metabolite, was included in this analysis. When AUC changes were not provided, the mean values for substrate and metabolite AUC were used for calculations. Similarly, if the M/P AUC ratios were not available, mean AUC values for metabolites and substrates were used to calculate the ratios. Additionally, parent/metabolite AUC ratios were converted to M/P AUC ratios, and changes in these ratios were calculated. For the correlation analysis, changes in substrate AUC, metabolite AUC, and M/P AUC ratio are presented as fold changes, with positive values indicating an increase and negative values indicating a decrease. If data were available from both an NDA and the literature for one study, data from the literature were used. Studies involving populations with altered enzyme activities (e.g., poor metabolizers) were excluded from this analysis.

## 3. Results

When all clinical DDI studies curated in the DIDB were considered, out of the 26,478 individual studies available, 7318 had at least one metabolite measured (28%). As shown in Table 1, when only index DDI studies were counted, a total of 3261 individual studies were available, with 1466 studies (45%) measuring at least one metabolite (the publications dates ranged from 1983 to 2024). Because one study may have multiple metabolites measured, the total number of metabolite entries was 1944. For consistency, the numbers for citations, DDI studies, and metabolite entries are called citation number, study entry number, and metabolite entry number, respectively. Among all the index DDI studies (*N* = 3261), 24% were performed using a cocktail approach (i.e., simultaneous administration of multiple index substrates), and 60% of these cocktail studies had metabolite data (versus 40% for non-cocktail studies). For the correlation analysis, a total of 466 metabolite entries for 144 precipitants had plasma measurements for both index substrates and metabolites and had parent substrate AUC changes ≥ 1.25-fold for inhibition or ≤0.8-fold for induction. Fold changes in AUC for index substrates and marker metabolites and M/P AUC ratios are presented here. A detailed review of the DDI studies with metabolite measurements for each index substrate is provided below and case studies are discussed.

### 3.1. CYP1A2 Caffeine

**Figure 1:**
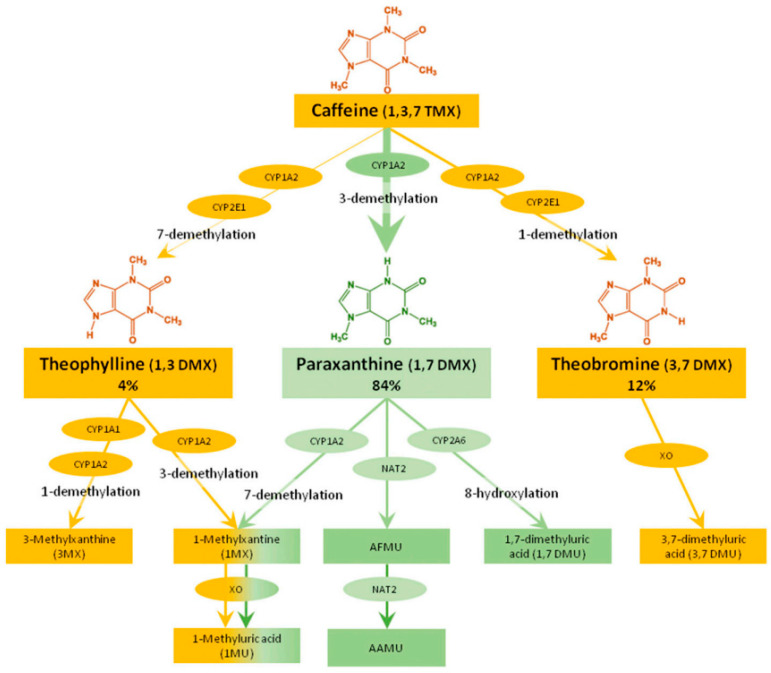
Main pathways and enzymes involved in caffeine degradation. The green pathway highlights paraxanthine formation, the primary metabolic pathway of caffeine [5].

Caffeine is the only recommended index substrate for CYP1A2 in the ICH M12 guidance [1]. Caffeine (1,3,7-trimethylxanthine) is rapidly and completely absorbed by the gastrointestinal tract and is extensively metabolized in the liver to form three active primary metabolites: paraxanthine (84%; 1,7-dimethylxanthine), theobromine (12%; 3,7-dimethylxanthine), and theophylline (4%; 1,3-dimethylxanthine). Caffeine is selectively metabolized by CYP1A2 to paraxanthine, while CYP2E1 also contributes to the formation of theobromine and theophylline. These three metabolites undergo further biotransformation by multiple enzymes. In addition to CYP1A1, CYP1A2, and CYP2A6, N-acetyltransferase-2 and xanthine oxidase contribute to the clearance of these three primary metabolites (Figure 1).

The plasma concentration of caffeine decreases more rapidly than that of paraxanthine. In humans, about 70% of an oral caffeine dose is recovered in urine, with ≤2% excreted unchanged. An almost complete (98%) reabsorption from the renal tubule after glomerular filtration indicates that metabolism is driving caffeine clearance [5].

Out of the 474 individual DDI studies available in the DIDB as of May 2024 evaluating oral caffeine as an object, 300 (63%) measured at least one metabolite. When considering only cocktail studies, 68% measured at least one caffeine metabolite (Table 1). Inhibition effects were studied more frequently than induction, representing 66% of all studies measuring at least one metabolite. Out of the studies including metabolite(s), paraxanthine was the most frequently measured (*N* = 210, 70%), as this metabolic pathway is specific to CYP1A2. The other two primary metabolites, theobromine and theophylline, were rarely used, with only nine and six metabolite entries, respectively. N-acetylated metabolites, 5-acethylamino-6-amino-3-methyluracyl (AAMU), and 5-acethylamino-6-formylamino-3-methyluracyl (AFMU) were measured on 44 occasions, while the xanthine oxidase pathway was reported on 42 occasions.

The earliest publication measuring caffeine metabolite(s) was published in 1986 and no trend was observed based on the publication date (Figure S1) [6]. Among drugs approved by the FDA in the past 10 years (Table 2), clinical DDI studies with caffeine were conducted for 16 drugs to investigate the inhibition or induction potential on CYP1A2, and five (31%) measured the metabolite paraxanthine.

Caffeine and metabolite/caffeine ratios were measured either in plasma/serum and/or urine. In a cocktail study investigating the potential drug interactions of tipranavir administered with low-dose ritonavir, Dumond et al. reported that both plasma and urinary measures of caffeine concentration indicated induction of CYP1A2, although the magnitude in the change from baseline differed with 47% decline for plasma caffeine AUC versus a 5% increase in the urinary ratio of [(1-methylxantine + 1-methyluric acid + AFMU)/1,7-dimethyluric acid] [7]. The authors hypothesized that the paraxanthine/caffeine plasma AUC ratio, not measured in their study, may have more closely mimicked the caffeine urinary ratio, as a previous report showed that urinary ratios correlated less strongly with CYP1A2 parameters in vitro than the plasma paraxanthine/caffeine AUC ratio [7]. Others recommend the use of either the plasma paraxanthine/caffeine AUC ratio or a more complex urinary ratio for estimation of CYP1A2 activity [8]. Multiple different urine metabolite ratios of caffeine have been used/proposed as probes for CYP1A2, and while some may be preferred as CYP1A2 activity markers, the plasma AUC ratio of paraxanthine/caffeine remains a more robust assessment [9].

**Figure 2:**
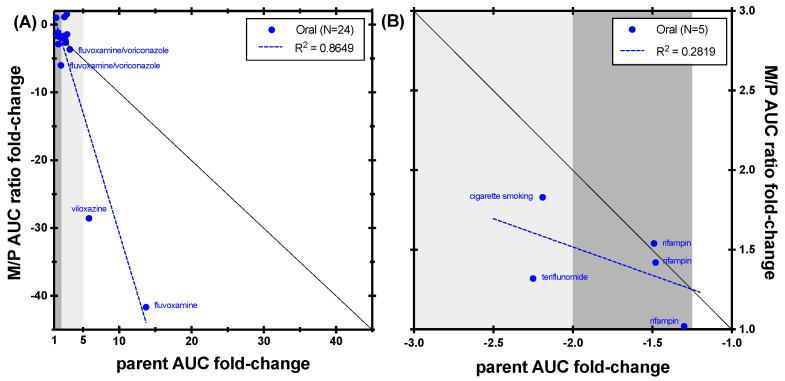
Correlation between caffeine AUC fold-change and paraxanthine/caffeine AUC ratio fold-change in (**A**) CYP1A2 inhibition studies and (**B**) CYP1A2 induction studies. The black line represents unity. Dark grey shading denotes (+/−) ≥ 1.25- to 2-fold change; light grey shading denotes (+/−) ≥ 2- to 5-fold change.

As shown in Figure 2, for a majority of CYP1A2 inhibitors caffeine AUC fold-change and paraxanthine/caffeine were similar. The M/P AUC ratio was more sensitive than the caffeine fold-change for a subset of drugs, with the biggest deviation from the unity line observed for the strong inhibitors viloxazine and fluvoxamine. For instance, upon co-administration of an oral dose of caffeine (200 mg) with the strong CYP1A2 inhibitor fluvoxamine 100 mg twice daily for 2 days, paraxanthine plasma AUC_0–24 h_ was decreased by 67% (*p* < 0.01), theobromine AUC_0–24 h_ increased 1.23-fold (not significant), and theophylline exposure increased 2.59-fold (*p* = 0.07) [10]. In contrast, the M/P AUC ratio was decreased by 98% for paraxanthine, 91% for theobromine, and 81% for theophylline, demonstrating the increased robustness of using M/P AUC ratio in contrast to metabolite formation changes alone.

Out of five induction studies with reportable M/P AUC ratios, teriflunomide (classified as a moderate inducer) showed a difference between the use of caffeine fold-change versus M/P AUC fold-change, with the later being less sensitive due to a decrease in paraxanthine plasma exposure following co-administration (Figure 2). Additional data would be needed to determine which endpoint is preferred for induction studies.

With 85% of the U.S. population consuming at least one caffeinated beverage per day, the quantifications of caffeine and its metabolites by liquid chromatography mass spectrometry require a considerable effort to select or find a surrogate matrix, without the compounds of interest, e.g., finding caffeine-free human plasma or urine volunteers may be challenging [11,12]. Even when subjects were asked to abstain from all caffeine-containing foods and beverages for 24 h prior to caffeine dose administration, Culm-Merdek et al. reported that predose plasma from nearly all subjects contained measurable concentrations of caffeine and paraxanthine [10]. Interference, linked to potential loss of sensitivity, may arise if the blank matrix used for calibration curves contains “endogenous” compounds from dietary caffeine intake [13]. Careful consideration should be given to the bioanalytical method development when using caffeine as an index substrate.

### 3.2. CYP2B6 Bupropion

**Figure 3:**
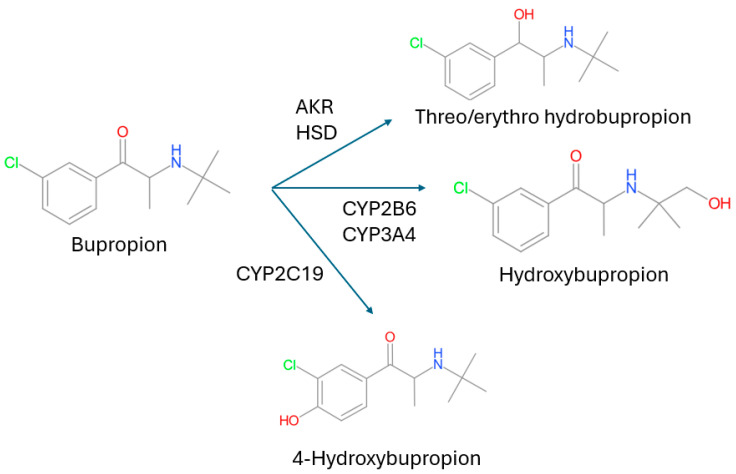
Biotransformation pathways of bupropion in vitro. AKR, aldo-keto reductase; HSD, 11-*β*-hydroxysteroid dehydrogenase 1.

Bupropion, an antidepressant medication also used in smoking cessation, has a complex metabolism. It is a racemic compound and is metabolized to hydroxybupropion predominantly by CYP2B6 and undergoes other hydroxylation reactions catalyzed by CYP2C19, and the ketone group of bupropion is reduced to give the erythrohydrobupropion and threohydrobupropion metabolites. The ketone reduction metabolism is catalyzed by 11-β-hydroxysteroid dehydrogenase and possibly other carbonyl reductases (Figure 3).

Out of the total 112 individual DDI studies evaluating oral bupropion as an object, 92 (82%) measured at least one metabolite *(*Table 1). When considering only cocktail studies with bupropion (*N* = 15), all studies measured a metabolite (Table 1).

The metabolites measured included hydroxybupropion (90 studies), erythrohydrobupropion (17 studies), and threohydrobupropion (18 studies). In five studies, the two reduced metabolites were measured but not separated. In contrast, more complex analytical methods have been used to separate the metabolite enantiomers in some studies. For example, in 15 studies the (R,R)- and (S,S)-hydroxy enantiomers were separated and in eight studies the (R,R)- and (S,S)-threohydrobupropion and (R,S)- and (S,R)-erythrohydrobupropion enantiomers were separated.

The timeline for publications of bupropion studies showed a large peak in 2022 due to a single study that measured 4 metabolites in subjects with different CYP2B6 genotypes giving a total of 72 metabolite measurements [14]. Over the past 10 years, 10 drugs approved by the FDA have included clinical DDI studies with bupropion to evaluate their inhibition or induction effects on CYP2B6. Hydroxybupropion was measured in studies for three drugs (30%), and (S,S)-hydroxybupropion was also measured in one of them.

Parent bupropion is a poor substrate for CYP2B6 activity as metabolism to the hydroxy metabolite was estimated to only account for 21% of a bupropion dose in a study by Sager et al. using in vitro metabolic systems [15]. Although there is some uncertainty to the exact amount of a dose of bupropion converted to hydroxybupropion (and downstream metabolites), interaction studies of bupropion with CYP2B6 inhibitors suggest that a larger proportion of the bupropion dose is metabolized by CYP2B6 or that the inhibitors also inhibit the alternative metabolic pathways of bupropion. Regardless of the precise amount of hydroxybupropion formation, by itself bupropion clearance is not a sensitive marker of CYP2B6, and it is necessary to measure hydroxybupropion in addition to the parent compound to gain an understanding of how a novel inhibitor/inducer will affect CYP2B6 activity [1].

However, even using the partial clearance of bupropion to hydroxybupropion as a marker activity for CYP2B6 has limitations. This is due to the (R,R)-hydroxybupropion isomer being the predominant isomer observed in humans. The PK of (R,R)-hydroxybupropion is elimination rate limited with the metabolite having a longer half-life than the parent compound. This limitation does not occur for the (S,S)-hydroxybupropion metabolite, and for this reason, the formation clearance of (S,S)-hydroxybupropion has been suggested as an improved measure of in vivo CYP2B6 activity compared to measurements based on racemic hydroxybupropion [16]. The (S,S)-hydroxybupropion metabolite was only measured in 15 of the DDI index studies. In one of these studies, (S,S)-hydroxybupropion formation was shown to be a more sensitive index marker than either racemic hydroxybupropion or (R,R)-hydroxybupropion for the increase in CYP2B6 caused by the effect of the CYP2B6 inducers rifampicin and ritonavir. However, due to insufficient data available, a correlation analysis was not performed for these enantiomers and their metabolites.

**Figure 4:**
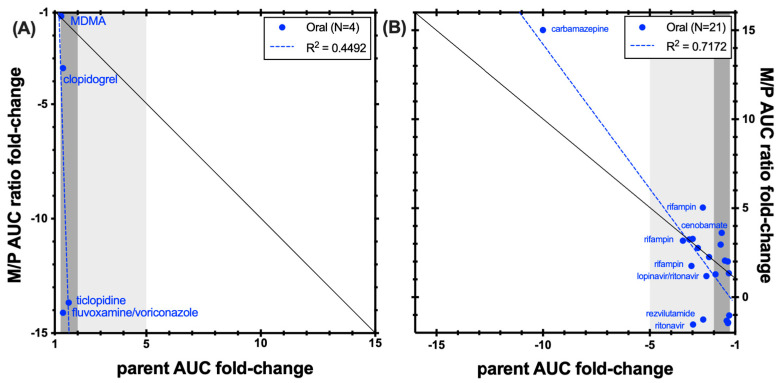
Correlation between bupropion AUC fold-change and hydroxybrupopion/bupropioon AUC ratio fold-change in (**A**) CYP2B6 inhibition studies and (**B**) CYP2B6 induction studies. The black line represents unity. Dark grey shading denotes (+/−) ≥ 1.25- to 2-fold change; light grey shading denotes (+/−) ≥ 2- to 5-fold change.

As shown in Figure 4, the hydroxybupropion M/P AUC ratio was dramatically reduced by inhibitors of CYP2B6 including clopidogrel, ticlopidine, and the combination of fluvoxamine and voriconazole, whilst the change in parent bupropion AUC in these studies was modest. Overall, these studies indicate that the M/P AUC ratio is a more sensitive indicator of CYP2B6 inhibition than changes in bupropion. This is illustrated for example in the study reported by Turpeinen et al. that showed that the CYP2B6 inhibitors ticlopidine and clopidogrel decreased the hydroxybupropion AUC significantly (61% and 89%, respectively), but bupropion clearance was only decreased by 26% and 36%, respectively [17]. Studies with prasugrel, a CYP2B6 inhibitor, showed bigger changes in the M/P AUC ratio compared with changes in either hydroxybupropion AUC or bupropion AUC when considered individually.

The picture is more complex when considering compounds that induce bupropion clearance. Several inducers, including carbamazepine, efavirenz, and rifampin, increased the M/P AUC ratio whilst decreasing the AUC of bupropion (Figure 3). Other inducers of bupropion clearance decreased both the M/P AUC ratio and the AUC of parent bupropion. This suggests that the induction of bupropion clearance may occur through pathways other than CYP2B6, or that the elimination of the hydroxy metabolite is also induced in addition to the effect of the inducer on bupropion hydroxylation by CYP2B6.

The impact of the CYP2B6 inducer efavirenz on the PK of bupropion and its hydroxy metabolite is complex. Multiple dosing of efavirenz resulted in the M/P AUC ratio increases of 2.3-fold, while the AUC of bupropion decreased by 55%. On chronic dosing, efavirenz changed the shape of the hydroxybupropion concentration-time profile and increased the AUC ratio of (S,S)-hydroxybupropion to (S)-bupropion ratio, confirming induction of CYP2B6. A single dose of efavirenz was shown to cause a decrease in hydroxybupropion exposure, suggesting inhibition of CYP2B6. It was also shown to have an effect on the elimination of (S,S)-threohydrobupropion. The effects of efavirenz were dependent also on the CYP2B6 genotype/phenotype of the individuals [14].

### 3.3. CYP2C8 Repaglinide

**Figure 5:**
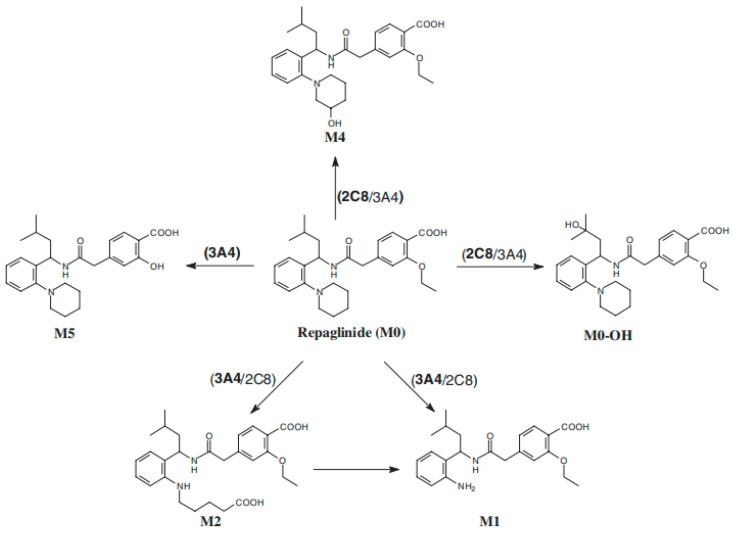
Biotransformation pathways of repaglinide in vitro. The principal enzyme responsible is highlighted in bold [19].

Repaglinide is indicated as an adjunct to diet and exercise to improve glycemic control in adults with type 2 diabetes mellitus [18]. CYP2C8 and CYP3A were found to be responsible for the conversion of repaglinide into its multiple metabolites [19,20]. In vitro studies with various systems suggest that formation of M0-OH (hydroxylated on the isopropyl moiety) and M4 (hydroxylation on the piperidine ring system) is predominantly mediated by CYP2C8, while the biotransformation of M1 (aromatic amine), M2 (oxidized dicarboxylic acid), and M5 is mainly mediated by CYP3A4, as shown in Figure 5 [19,20]. The repaglinide M4 metabolic pathway has been proposed as a specific CYP2C8 probe for the assessment of DDIs [20]. Metabolites do not contribute to the glucose-lowering effect of repaglinide [18]. Based on the highest AUC ratio observed with co-administration of gemfibrozil 900 mg single dose, a strong CYP2C8 inhibitor, the fraction metabolized by CYP2C8 was estimated to be 0.88 [21]. However, this value may overestimate that fraction as repaglinide is also a substrate of the organic anion-transporting polypeptide (OATP)1B1 and OATP1B3 and gemfibrozil (and its glucuronide metabolite) inhibit these transporters. Following single-dose oral administration, approximately 90% of the radiolabel was recovered in the feces and approximately 8% in the urine (0.1% as the parent), and the major metabolite M2 accounted for 60% of the administered dose [18].

There were a total of 132 DDI study entries measuring repaglinide, including 38 studies (29%) measuring at least one metabolite (Table 1), where repaglinide was all orally administered. Among the 38 studies, 24 studies were with gemfibrozil, a strong CYP2C8 inhibitor, and 3 studies with clopidogrel, a moderate CYP2C8 inhibitor.

A total of 4 metabolites were measured, including M0-OH, M1, M2, and M4, making up to 102 metabolite entries (99 from plasma and 3 from urine). All these metabolite entries were obtained from inhibition DDI studies available in 12 citations published from 2003 to 2015. M1 was measured in all the citations, M2 and M4 were measured in nine of them, while only one citation measured M0-OH. Chronologically, there seems to be a peak in metabolite entries around 2010, with 66 metabolite entries (65%) obtained from five citations published in 2008, 2009, and 2011 when 3–4 metabolites were measured simultaneously in these studies (Figure S1). Regarding the study approach, only six studies were conducted in cocktail and none of them had metabolite measured (Table 1). As for drugs approved recently, clinical DDI studies with repaglinide were performed for 11 drugs to investigate the inhibition or induction potential of CYP2C8. However, none of these clinical DDI evaluated any metabolites of repaglinide.

Most of the DDI studies were related to known CYP2C8 inhibitors. Clopidogrel, gemfibrozil, and trimethoprim were studied in 10 citations. In these DDI evaluations, M1 was measured for all of them, whereas M4, although proposed as a specific pathway for CYP2C8, was measured in only seven citations. Following a clinical dose of 600 mg twice daily administration of gemfibrozil, a strong CYP2C8 inhibitor, in healthy subjects, repaglinide (administered 1 h after the last dose of gemfibrozil) AUC was increased 6.98- to 8.22-fold. The AUC for M4 was decreased by 77–89%. A more dramatic change in the M4/parent AUC ratio was observed, with decreases of 95–97%, supporting the predominant role of CYP2C8 in the transformation of M4 [22,23,24,25]. Because CYP2C8 was inhibited by gemfibrozil, the metabolism of repaglinide was shifted to CYP3A, leading to increased formation of M1 and M2. In these studies, the AUC of M1 was found to increase 3.40- to 4.62-fold, and M2 1.30- to 2.06-fold; however, the M/P AUC ratio for M1 was decreased, with respective decreases up to 58% and 82%.

**Figure 6:**
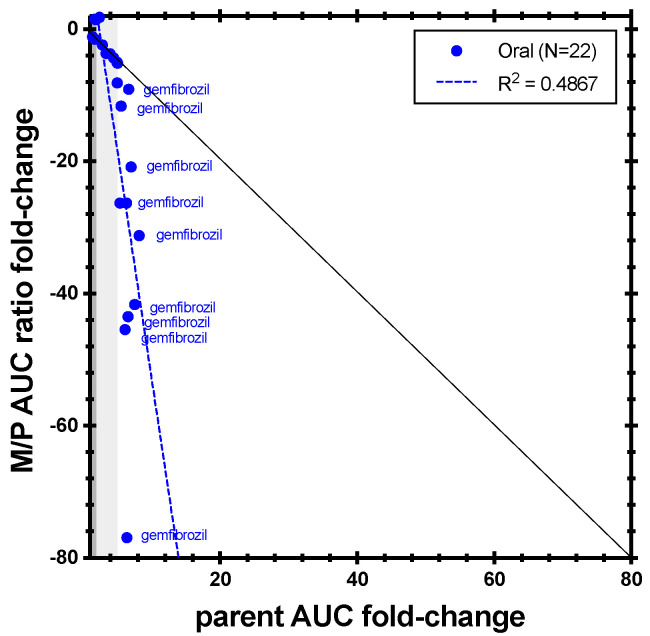
Correlation between repaglinide AUC fold-change and M4/repaglinide AUC ratio fold-change in CYP2C8 inhibition studies. The black line represents unity. Dark grey shading denotes (+/−) ≥ 1.25- to 2-fold change; light grey shading denotes (+/−) ≥ 2- to 5-fold change.

The correlation analysis between repaglinide and M4 metabolite (*N* = 22; Figure 6) showed no overall correlation. However, when reviewing the data based on potency, the results varied. For weak and moderate CYP2C8 inhibition by clopidogrel or gemfibrozil (repaglinide AUC increases of 1.42- to 4.98-fold), the M/P AUC ratio changes (1.15- to 5-fold decrease) appeared similar to repaglinide AUC changes. For strong inhibition (repaglinide AUC increases of 5- to 8.26-fold), the M/P AUC ratio changes were more pronounced, with decreases of 5.15- to 76.92-fold. Although a few drugs showed increases in M4 AUC, the M/P AUC changes for M4 decreased for all except cyclosporine and telithromycin, both CYP3A inhibitors.

### 3.4. CYP2C9 (S)-Warfarin and Flurbiprofen

#### 3.4.1. (S)-Warfarin

Warfarin is a vitamin K antagonist indicated for the prophylaxis and treatment of venous thrombosis and thromboembolic diseases. Warfarin is administered as a racemic mixture. (S)-warfarin is metabolized primarily by CYP2C9 to (S)-7-hydroxywarfarin, an inactive major metabolite [26,27]. Studies with radiolabeled drugs have demonstrated that up to 92% of the orally administered dose is recovered in urine. Very little warfarin is excreted unchanged in urine. Urinary excretion is in the form of metabolites [28].

A total of 390 DDI study entries measured (S)-warfarin (all following oral dosing of racemic warfarin), including 41 studies (11%) measuring metabolite (Table 1). Among the 41 DDI studies with metabolite measured, 30 were inhibition studies, 9 were induction studies, and 2 were mediated by other mechanisms. Regarding the cocktail approach, there were 55 cocktail studies, and 10 of them had metabolite measured (Table 1). Overall, the DDI studies were obtained from 30 publications and three NDAs. Among them, more than 1/3 of articles were published prior to 2000 (*N* = 11), while 10 articles were published in the past 10 years (Figure S1). No chronological trend was observed regarding the metabolite measurement. Additionally, among drugs approved in the past 10 years, clinical DDI studies with (S)-warfarin were performed for 24 drugs, yet none measured warfarin metabolite.

A total of 13 metabolites were quantified from 81 metabolite entries, with (S)-7-hydroxywarfarin being the most measured (*N* = 32), followed by (S)-6-hydroxywarfarin (*N* = 14), (S)-8-hydroxywarfarin (*N* = 9), and 7-hydroxywarfarin (*N* = 8). However, most metabolite entries only contained clearance or excretion (in urine and stool) data. Only two entries showed positive inhibition and two showed positive induction with plasma AUC data for (S)-warfarin and its hydroxy metabolite, and the two induction entries did not have the M/P AUC ratio change values, thus correlation analysis could not be performed. Given the limited data, the metabolite measurements did not appear to enhance the understanding of the mechanism.

#### 3.4.2. Flurbiprofen

Flurbiprofen is a nonsteroidal anti-inflammatory drug indicated for relief of the signs and symptoms of rheumatoid arthritis and osteoarthritis [29]. In vitro studies have demonstrated that CYP2C9 may be the only CYP isoform involved in the metabolism of flurbiprofen to its major metabolite 4′-hydroxyflurbiprofen [30,31]. UGT2B7 is the predominant UGT isozyme responsible for the further glucuronidation of both (R)- and (S)- enantiomers of flurbiprofen and 4′-hydroxyflurbiprofen [32,33]. The 4′-hydroxyflurbiprofen metabolite showed little anti-inflammatory activity in animal models of inflammation. Following oral administration, less than 3% of flurbiprofen was excreted unchanged in the urine, with about 70% of the dose eliminated in the urine as flurbiprofen, 4′-hydroxyflurbiprofen, and their acylglucuronide conjugates [29]. No clinical studies with CYP2C9 strong inhibitors were performed. Based on the maximum AUC ratio observed with fluconazole (400 mg once daily for 7 days), a CYP2C9 moderate inhibitor, the estimated fraction metabolized by CYP2C9 was at least 0.67 [34]. Thus, flurbiprofen could be considered a moderate sensitive substrate of CYP2C9 based on the data available.

A total of 47 DDI study entries measuring flurbiprofen (all following oral dosing), including 40 studies (85%) measuring metabolite (Table 1). Only one metabolite, 4′-hydroxyflurbiprofen was measured. Among the 40 DDI studies with metabolite measured, 33 were inhibition studies and 7 were induction studies obtained from 23 citations. The majority of the citations were published prior to 2020 (*N* = 20), including 8 from 2001 to 2010 and 12 from 2011 to 2020. No chronological trend was observed regarding the metabolite measurement (Figure S1). There were 20 cocktail studies and 75% of them measured 4′-hydroxyflurbiprofen (Table 1).

**Figure 7:**
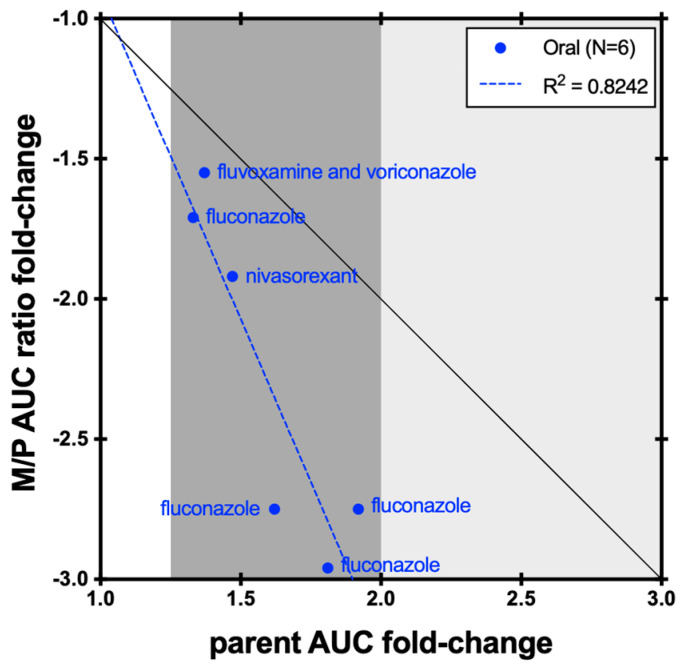
Correlation between flurbiprofen AUC fold-change and 4′-hydroxyflurbiprofen/flurbiprofen AUC ratio fold-change in CYP2C9 inhibition studies. The black line represents unity. Dark grey shading denotes (+/−) ≥ 1.25- to 2-fold change; light grey shading denotes (+/−) ≥ 2- to 5-fold change.

For inhibition studies (*N* = 33), 15 studies (45%) showed positive results by fluconazole, a moderate CYP2C9 inhibitor. Following co-administration with fluconazole 200 mg for 2 doses (*N* = 3), flurbiprofen AUC was increased 1.62- to 1.92-fold whereas the AUC of 4′-hydroxyflurbiprofen was reduced by 30–41%. A larger decrease of 63.7–66.2% was observed in the M/P AUC ratio, suggesting a more sensitive marker to reflect the PK changes (Figure 7) [35,36,37]. Despite the limited dataset (*N* = 6), a correlation between flurbiprofen AUC and M/P AUC ratio was observed (R^2^ = 0.8242, Figure 7).

For induction studies (*N* = 7), four studies showed positive results by rifampin and metamizole, both inducers of multiple CYPs including CYP2C9. Co-administration of flurbiprofen (including 1 cocktail study) with rifampin (600 mg once daily for 5–7 days) caused a 26–32% decrease in the AUC of flurbiprofen. Only one study had the AUC value available for 4′-hydroxyflurbiprofen and there was a 10% increase in the presence of rifampin. The 4′-hydroxyflurbiprofen M/P AUC ratio was increased 1.23- to 1.47-fold [38,39]. Following concomitant administration with metamizole (1 g three times daily for 7 days), flurbiprofen AUC was reduced by 20–22%, and 4′-hydroxyflurbiprofen AUC was also reduced by 12–24%, while M/P AUC ratio was increased 1.11- to 1.13-fold [40,41]. Based on the small dataset with weak induction, the M/P AUC ratio does not seem to be a more sensitive indicator than flurbiprofen.

More than half of the studies showed no effect on the exposure of flurbiprofen. A further review of the PK data for 4′-hydroxyflurbiprofen did not add value to the interpretation of the results, although relatively larger changes (but still below the threshold) in the AUC of 4′-hydroxyflurbiprofen or 4′-hydroxyflurbiprofen M/P AUC ratio were observed in two studies with St. John’s wort [42].

Compared to (S)-warfarin, flurbiprofen was less commonly used as a CYP2C9 substrate in the DDI evaluation during drug development. Only three drugs approved in the past 10 years were studied with flurbiprofen to investigate the inhibition or induction potential on CYP2C9 and none of them showed an effect on the exposure of flurbiprofen. Among them, 4′-hydroxyflurbiprofen PK was measured only for one drug.

### 3.5. CYP2C19 Omeprazole

Omeprazole, a proton-pump inhibitor, is the only index substrate recommended in ICH M12 for the polymorphic enzyme CYP2C19. The PK of omeprazole has high interindividual variability. As well as undergoing 5-hydroxylation mediated predominantly by CYP2C19, omeprazole is also metabolized to a sulfone metabolite by CYP3A4. The metabolites are then further metabolized to give the hydroxysulfone metabolite. Sulfoxidation of the 5-hydroxy metabolite is presumably by CYP3A4 whilst the sulfone is hydroxylated by CYP2C19. In subjects that are poor metabolizers for CYP2C19, sulfoxidation by CYP3A4 is the major metabolic pathway, although the formation of 5-hydroxyomeprazole has also been observed. The latter observation together with data showing that clarithromycin (a CYP3A4 inhibitor) can decrease 5-hydroxyomeprazole formation in CYP2C19 poor metabolizer subjects suggest a role for CYP3A4 in 5-hydroxyomeprazole formation.

For omeprazole, 331 DDI studies were reported in the DIDB of which 143 were cocktail studies. Metabolites were measured in 201 studies and in 95 cocktails studies (Table 1). This equates to metabolites being measured in 61% of all studies and in 66% of the cocktail studies. In addition, 2 studies evaluated DDIs with (R)-omeprazole and 20 studies with esomeprazole (the S-isomer). Metabolites were measured in both (R)-omeprazole studies and in four of the studies with esomeprazole (20%). The 5-hydroxyomeprazole metabolite was measured more frequently than the sulfone by a ratio of approximately 3:1. The 5-hydroxyomeprazole (CYP2C19 pathway) was measured on 195 occurrences, while omeprazole sulfone (CYP3A4 pathway) was included in 65 metabolite entries (inhibition and induction studies).

The omeprazole metabolite measurements did not show an obvious trend with time over the period 2003–2023 (Figure S1). Among drugs approved in the past 10 years, a total of 20 drugs had clinical DDI studies with omeprazole to assess the effect on CYP2C19. Not surprisingly, metabolites were not commonly measured in these studies. Only four drugs (20%) had 5-hydroxyomeprazole measured (Table 2).

**Figure 8:**
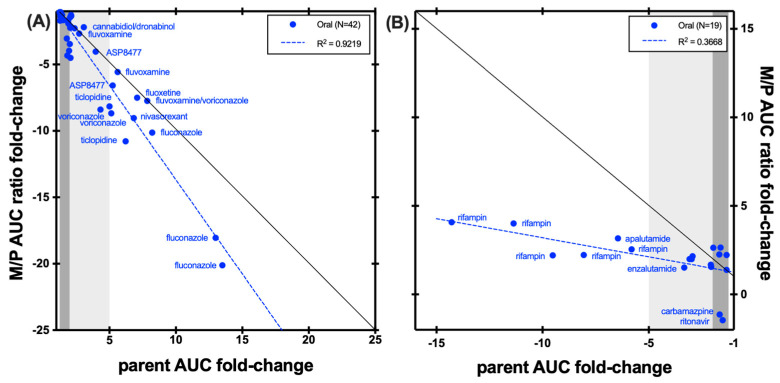
Correlation between omeprazole AUC fold-change and 5-hydroxyomeprazole/omeprazole AUC ratio fold-change in (**A**) CYP2C19 inhibition studies (**B**) CYP2C19 induction studies. The black line represents unity. Dark grey shading denotes (+/−) ≥ 1.25- to 2-fold change; light grey shading denotes (+/−) ≥ 2- to 5-fold change.

As shown in Figure 8, there is a strong correlation between the fold-change in the 5-hydroxyomeprazole/omeprazole plasma AUC ratio and the change in parent omeprazole AUC when the action of CYP2C19 inhibitors is considered. Whilst this suggests that similar conclusions can be drawn from measuring only the parent compound, measurement of the 5-hydroxyomeprazole/omeprazole AUC ratio has been used to help normalize the variability associated with the absorption and metabolism of omeprazole. This is exemplified in the study reported by Haberer et al., with a within-subject variability (*N* = 21 subjects) of AUC_0-inf_ of 54.3% and this was reduced to 13.7% using the 5-hydroxyomeprazole/omeprazole AUC ratio [43].

Due to being metabolized by CYP2C19 and CYP3A4, the levels of the two primary metabolites may be monitored to allow differentiation of the changes in the two enzymes as a cause of any observed drug interaction. For instance, carbamazepine induced omeprazole clearance and increased the levels of the sulfone. Concomitantly, the levels of the 5-hydroxyomeprazole metabolite (catalyzed by CYP2C19) were decreased. In this study, the sulphone metabolite to omeprazole AUC ratio increased, although not to a statistically significant level, probably due to the small number of subjects in the study (*N* = 5). However, there was no change in the 5-hydroxyomprazole to omeprazole AUC ratio. Overall, the data suggest that carbamazepine induces CYP3A4 to a greater extent than it induces CYP2C19.

In contrast to carbamazepine where the omeprazole and 5-hydroxyomeprazole were decreased to the same extent (AUC ratio of 5-hydroxyomeprazole/omeprazole in the presence of carbamazepine ~1), other inducers such as rifampin caused a bigger decrease in the AUC of omeprazole than 5-hydroxyomeprazole, such that M/P ratio was consistently >1 (Figure 8). These results were consistent with simulations, where carbamazepine induced only CYP3A4 whilst rifampin induced both CYP3A4 and CYP2C19 but with a bigger effect on CYP3A4.

### 3.6. CYP2D6 Desipramine, Dextromethorphan, and Nebivolol

#### 3.6.1. Desipramine

Desipramine, an antidepressant, was used in 81 DDI studies. Metabolites were measured in 43 of these studies (53%) (Table 1). The major metabolite measured for desipramine is the 2-hydroxy (45 study entries), while the 10-hydroxy (2 entries), 2-hydroxydespramine glucuronide (2 entries) and 10-hydroxydesipramine glucuronide (1 entry) metabolites were generally not included. No cocktail studies were conducted with desipramine (Table 1). Looking at the years of publication, no DDI studies with desipramine metabolite measured have been published since 2015, and there was no chronological trend over the years from 1984 to 2015 (Figure S1). Although desipramine is recommended as a CYP2D6 index substrate, it has not been commonly used to measure CYP2D6 activity during drug development. Among drugs approved in the past 10 years, only 2 drugs included DDI studies with desipramine. Neither study measured any metabolites.

**Figure 9:**
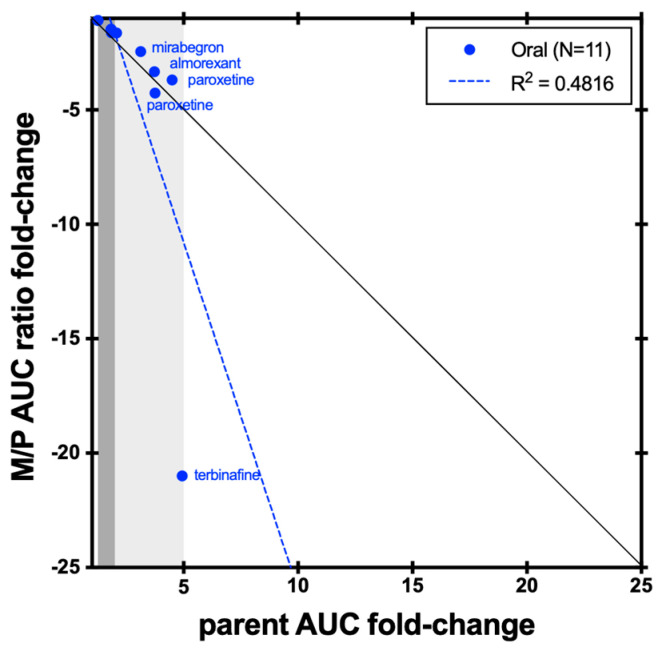
Correlation between desipramine AUC fold-change and 2-hydroxydesipramine/desipramine AUC ratio fold-change CYP2D6 inhibition studies. The black line represents unity. Dark grey shading denotes (+/−) ≥ 1.25- to 2-fold change; light grey shading denotes (+/−) ≥ 2- to 5-fold change.

The conversion of desipramine to 2-hydroxydesipramine is catalyzed by CYP2D6. As shown in Figure 9, except terbinafine, the changes in 2-hydroxydesipramine/desipramine AUC ratio in the presence of a CYP2D6 inhibitor, regardless of potency, were similar in magnitude to those observed for desipramine. This is likely due to the relatively high fraction metabolized by CYP2D6 for desipramine in subjects who express the enzyme (~80% on average). For terbinafine, the M/P AUC ratio is more sensitive to CYP2D6 inhibition than the change in parent desipramine.

#### 3.6.2. Dextromethorphan

**Figure 10:**
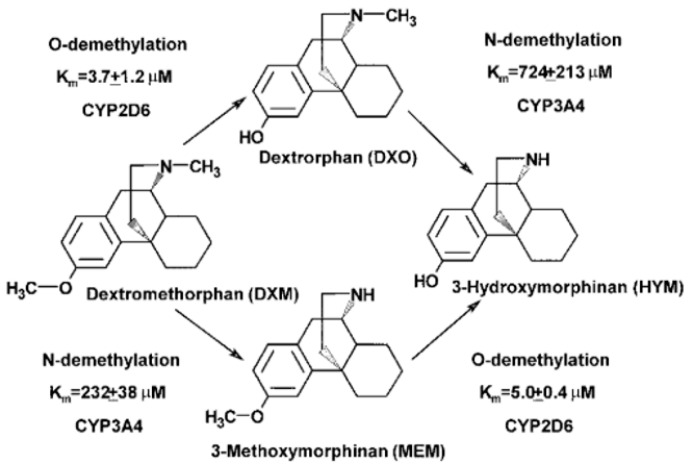
Dextromethorphan demethylation pathways catalyzed by recombinant human CYP2D6 and CYP3A4 [44].

Dextromethorphan is a widely used antitussive agent present in several over-the-counter medications. Dextromethorphan is subject to *O*-demethylation via CYP2D6 to form dextrorphan, a major active metabolite, and *N*-demethylation primarily via CYP3A4 to produce 3-methoxymorphinan. Both of these primary metabolites are further transformed to the demethylated 3- hydroxymorphinan, as shown in Figure 10 [44]. Major urinary excretion products of dextromethorphan are glucuronide conjugates of dextrorphan and 3-hydroxymorphinan [45]. CYP2D6 is a polymorphic enzyme, and as such, dextromethorphan half-life varies from approximately 4 h in extensive metabolizers to 30 h in poor metabolizers.

Out of the total 263 individual DDI studies evaluating oral dextromethorphan as an object, 215 (82%) measured at least one metabolite. When considering only cocktail studies (*N* = 106), 78 measured at least one metabolite (74%). Since CYP2D6 is not considered inducible, 85% of the studies investigated inhibition, but dextromethorphan was still included in induction studies, either using the dextrorphan/dextromethorphan ratio as a “negative” control for CYP2D6 or investigating CYP3A induction using the 3-methoxymorphinan/dextromethorphan ratio. For example, the effect of dicloxacillin on dextromethorphan was investigated as part of a cocktail study; while dicloxacillin caused a 48% decrease in dextromethorphan AUC, since the amount of dextrorphan in urine/dextromethorphan AUC was not impacted, the change in the object AUC was attributed to CYP3A induction [46].

From the studies including metabolite(s), dextrorphan was the most frequently measured (*N* = 205, 95%), while the other primary metabolite, 3-methoxymorphinan, was reported in only 33 (15%) of these studies. The secondary metabolite, 3-hydroxymorphinan followed with 12 study entries. Measurement of dextrorphan glucuronide or 3-hydroxymorphinan glucuronide was a rare occurrence (*N* = 5).

The earliest publication measuring dextrorphan was published in 1990 (Figure S1). There was no chronological trend regarding the metabolite measurement in the literature. Dextromethorphan was used as a substrate for CYP2D6 activity in clinical trials for 11 drugs approved in the past 10 years (Table 2). Of these trials, four (36%) measured dextrorphan levels, including three using plasma samples and one using urine samples.

**Figure 11:**
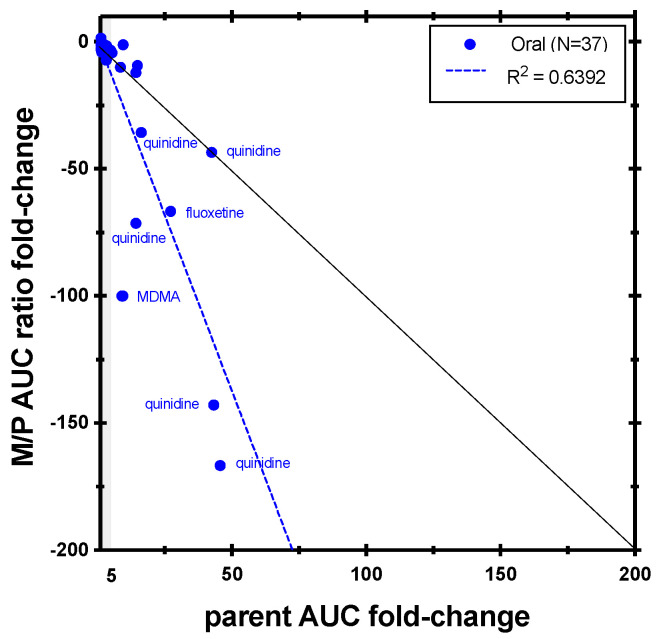
Correlation between dextromethorphan AUC fold-change and dextrorphan/dextromethorphan AUC ratio fold-change in CYP2D6 inhibition studies. The black line represents unity. Light grey shading denotes (+/−) ≥ 2- to 5-fold change.

Reports of metabolic ratio are still a current practice. As shown in Figure 11, the dextrorphan/dextromethorphan AUC ratio is a more sensitive endpoint to measure CYP2D6 inhibition in contrast to the parent AUC change alone. From the studies monitoring dextrorphan with a positive inhibition effect, quinidine was the most frequently used precipitant (the only precipitant reported in four different studies), including a multiple-dose study in healthy subjects with CYP2D6 extensive metabolizers evaluating the profile of a selected fixed-dose combination of quinidine with dextromethorphan [47]. A combination product of dextromethorphan and quinidine was later approved for the treatment of pseudobulbar effect [48].

#### 3.6.3. Nebivolol

Nebivolol was used in 11 studies with metabolites being measured in 7 (64%) studies. In addition, 13 studies were reported for d- and l-nebivolol enantiomers, in none of these studies were metabolites measured (Table 1). In addition to metabolism via N-dealkylation and oxidation by CYP2D6, nebivolol is also metabolized via direct glucuronidation. No cocktail studies were conducted with nebivolol or the enantiomers (Table 1). No drugs approved in the past 10 years have used nebivolol to measure CYP2D6 activity (Table 2).

The metabolites measured for nebivolol included 4-hydroxynebivolol (measured in four studies) with occasional studies reporting measurement of glucuronide metabolites. The metabolism of nebivolol to 4-hydroxynebivolol has been shown to be catalyzed by CYP2D6 [49]. The hydroxylated metabolites were primarily measured as they contribute to the pharmacodynamic activity of nebivolol. Due to the scarcity of information available, the correlation analysis between parent and metabolite measurements was not conducted.

### 3.7. CYP3A Midazolam and Triazolam

#### 3.7.1. Midazolam

The short-acting benzodiazepine midazolam, used therapeutically for sedation, anxiolysis, and amnesia, is the index substrate of choice for the evaluation of CYP3A-mediated DDIs. While midazolam clearance after oral administration reflects combined hepatic and intestinal CYP3A activity, systemic midazolam clearance after intravenous (IV) administration can be used to specifically characterize the hepatic contribution of CYP3A activity. Midazolam is not a substrate of major transporters at therapeutic concentrations; in particular, it is not a substrate of the efflux transporter P-glycoprotein [50].

Midazolam is extensively metabolized and is cleared primarily via 1′-hydroxylation (75%) and 4-hydroxylation (4%) by CYP3A, with an estimated fraction metabolized over 90% [51,52]. The main urinary excretion products of midazolam are glucuronide conjugates of the hydroxylated derivatives, formed by UGT2B4 and UGT2B7 [53,54]. Conversion to the glucuronides is rapid and plasma levels of unconjugated hydroxy midazolam metabolites are generally low with probe doses. The amount of midazolam excreted unchanged in the urine after a single IV dose is less than 0.5% [51]. In addition to CYP3A-mediated hydroxy metabolites, an additional primary metabolite was identified more recently, midazolam N-glucuronide, formed predominantly by UGT1A4, and accounting for 1–2% of the dose [53,55]. Midazolam’s clinical activity is due to both the parent drug and its 1′-hydroxy metabolite, which was found to have a significant pharmacological activity [56,57].

Midazolam dataset extracted from the DIDB was the largest of all the index substrates. Out of a total of 1283 individual DDI studies evaluating midazolam as an object drug, 468 (36%) measured at least one metabolite. When considering only cocktail studies (*N* = 241), 132 measured at least one midazolam metabolite (55%). Overall, five metabolites were measured, including the three primary metabolites (1′-hydroxymidazolam, 4-hydroxymidazolam, and midazolam N-glucuronide), and the two secondary glucuronides. Most studies measuring midazolam metabolites measured the 1′-hydroxymidazolam (90%), mainly in plasma, with only 7% measuring the 4-hydroxy metabolite. Glucuronide metabolites were rarely measured and only two recent, mechanistic evaluations by the same research team measured the more recently identified midazolam N-glucuronide [53,58]. The earliest publication measuring midazolam metabolite(s) was published in 1996 and no clear trend was observed based on the publication date (Figure S1). When evaluating recent NDAs, 100 drugs had dedicated clinical studies using midazolam to assess the effect of potential perpetrators on CYP3A (midazolam was given orally in 97% of cases). Of these, 35 drugs (35%) measured 1′-hydroxymidazolam levels (Table 2), a number similar to that observed in the literature data (Table 1). Out of the 515 metabolite entries, the majority were part of inhibition studies (*N* = 343, 67%). When considering midazolam route of administration, most of the clinical evaluations used oral midazolam (83% and 76% of inhibition and induction studies, respectively).

**Figure 12:**
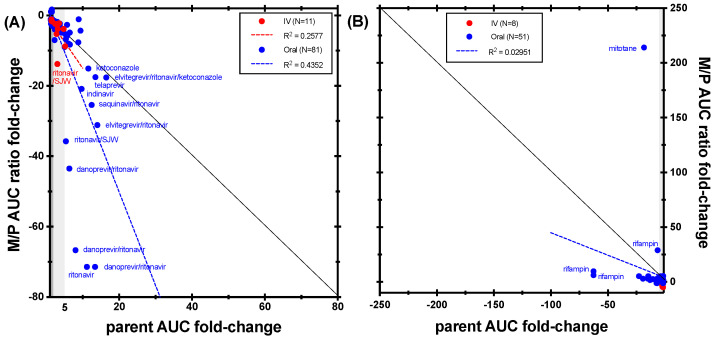
Correlation between midazolam AUC fold-change and 1′-hydroxymidazolam/midazolam AUC ratio fold-change in (**A**) CYP3A inhibition studies and (**B**) CYP3A induction studies. The black line represents unity. Dark grey shading denotes (+/−) ≥ 1.25- to 2-fold change; light grey shading denotes (+/−) ≥ 2- to 5-fold change.

Positive results with a plasma AUC value available for both midazolam and its 1′-hydroxy metabolite were considered in the correlation analysis. While one might expect that the AUC of 1′-hydroxymidazolam will likely decrease in case of inhibition because of decreased formation, the results show that in many cases, the metabolite exposure in fact increased and that this was independent of the inhibition potency (Figure S3). When the M/P AUC ratio is considered, as shown in Figure 12 for inhibition studies (*N* = 92), the results are mostly consistent with expectations (decreased metabolic ratio in the case of inhibition), but no clear correlation between the fold-change in M/P AUC ratio and midazolam AUC was found (R^2^ = 0.4352 and 0.2577 for oral and IV midazolam, respectively). Interestingly, for the most potent perpetrators, leading to over 10-fold change in midazolam AUC, the M/P AUC ratio seems notably more sensitive to the interaction than the change in the parent midazolam AUC (Figure 11 and Figure S2). However, these very large changes in the metabolic ratio were mostly observed with ritonavir and protease inhibitors containing ritonavir and might be explained by a more complex interaction mechanism, with mixed inhibition and induction of both midazolam and 1′-hydroxylation clearance, as well as possible induction of glucuronidation, and/or by saturation of metabolite formation at very high parent concentrations [53,59,60,61]. These cases illustrate the challenges in interpreting the metabolite information in the presence of additional mechanisms that might affect metabolite clearance. For induction studies (Figure 11; *N* = 59), no correlation was found, and in most cases, the parent AUC fold-change seemed to be more sensitive than the M/P AUC ratio.

Taken together, these results suggest that, while midazolam changes in AUC represent a reliable and sensitive measure to evaluate CYP3A perpetrators’ potency, the changes in 1′-hydroxymidazolam levels can be more complex to analyze. This confirms the observations made recently by Magliocca et al. on a small set of in-house CYP3A DDI studies with midazolam and might be partially explained by the generally low levels of circulating 1′-hydroxymidazolam and its rapid conjugation, making the measurement of the change in metabolite challenging, but also by changes in the metabolite clearance in case of additional mechanism(s) of interaction [4]. If the goal of the DDI study is to assess the potency of a CYP3A perpetrator, then measuring only midazolam changes in exposure after oral administration with the perpetrator seems to be an acceptable approach. Measuring the 1′-hydroxy metabolite, however, could be considered if the underlying DDI mechanism might be more complex or if measuring the total active moiety (parent and active metabolite) is important for the evaluation of the DDI clinical outcome.

#### 3.7.2. Triazolam

Only a small number of studies were found in the DIDB with triazolam as a victim drug (*N* = 61), confirming that midazolam is the preferred index substrate used to measure changes in CYP3A activity, with metabolite measurement (α-hydroxytriazolam) in only 8% of cases. No drugs approved in the past 10 years have used triazolam to measure CYP3A4 activity. Because of the limited amount of information available, a detailed analysis of the relationship between parent and metabolite measurements was not possible.

## 4. Discussion and Conclusions

In this review, an analysis was performed to evaluate metabolite measurement in clinical index DDI studies obtained from the literature and recent NDA reviews. The evaluation of metabolite(s) among clinical DDI studies with index substrates varied from a mere 11% when using (S)-warfarin to more than 80% for bupropion, flurbiprofen, and dextromethorphan. While a higher occurrence of metabolite measurement for bupropion and dextromethorphan could be explained by their more complex metabolism (≥2 primary pathways), this is not the case for flurbiprofen. Except for midazolam, measurement of metabolites was less frequent in the subset of studies included in the recent NDA submissions. A trend was observed towards a higher inclusion of metabolite(s) measurement in cocktails studies versus single index substrate, likely due to the default inclusion of metabolite(s) in relatively common cocktails, such as the “Cooperstown” cocktail.

An exploratory analysis was conducted to compare plasma exposure changes of the parent index substrate, marker metabolite, and M/P ratios observed in positive clinical DDI studies. Measuring metabolite can be important for certain index substrates. For a few substrates, e.g., caffeine (CYP1A2), bupropion (CYP2B6), and dextromethorphan (CYP2D6), measurement of metabolite and the use of M/P AUC ratios seems to provide more sensitivity to the effect of DDI perpetrators than using changes in the substrate PK itself. In contrast, changes in metabolite AUC alone are not a robust endpoint. For omeprazole (CYP2C19), the use of the M/P AUC ratio has been shown to help normalize the variability associated with the absorption/metabolism of the probe. Therefore, for these probes, the inclusion of primary metabolite measurement would be expected to refine the outcome of the clinical DDI studies. For midazolam (CYP3A), the added value of measuring the M/P AUC ratio was not as clear.

The inclusion of metabolite measurement can also provide mechanistic insights to understand complex mechanisms. For example, repaglinide, an index substrate of CYP2C8, is also metabolized by CYP3A and is a substrate of the transporters OATP1B1 and OATP1B3. Measuring CYP-specific metabolites is necessary to understand the DDI mechanism if the perpetrator inhibits these enzymes and transporters. Therefore, scientific judgment in study design is warranted. Of note, measurement of metabolite when using bupropion and omeprazole is suggested in the ICH M12 guidance, but it is not specifically mentioned for other index substrates. Finally, when metabolites significantly contribute to the clinical activity (nebivolol, midazolam), measuring the total active moiety may provide useful insight regarding the potential clinical outcome of the interaction if the index drug is likely to be co-administered with the perpetrator.

Additionally, practical analytical and bioanalytical challenges in measuring substrates and metabolites in clinical studies must be addressed carefully, for example, issues such as the separation of enantiomers in bupropion metabolites, low quantification levels of 1′-hydroxymidazolam in circulation, quantification of multiple metabolites using a cocktail approach, and caffeine levels in blank samples need to be considered. It is essential to use an adequate, highly sensitive, and robust bioanalytical method for each metabolite. Appropriate sample collection and storage should also be considered to avoid degradation of analytes.

Overall, the decision to measure the marker metabolite for a clinical index DDI study should be evaluated on a case-by-case basis. This evaluation should consider multiple factors, such as the inhibition or induction properties of the precipitant, the possibility of concomitant use of the index substrate, the pharmacological effects of the metabolite, and the analytical sensitivity required for accurate measurement of the metabolites.

There are limitations to this analysis. One significant hurdle was that the M/P AUC ratios, which were a key metric for the correlation analysis, were not consistently provided across studies, and sometimes not provided at all. In these cases, we had to calculate the ratios using the mean values of the metabolite and parent AUC provided in the PK analysis. Similarly, for parent and metabolite AUC changes, if the geometric mean ratios were not available in the publication or NDA review, the AUC changes were calculated based on mean values as well. For this reason, comparing the intersubject variability observed for the different measurements was not possible.

Additionally, because the aim of this analysis was to obtain an overall evaluation of the use of metabolite measurement in index DDI studies, all clinical DDI studies available in the DIDB were included, without using strict selection criteria. Some older studies, however, might not have reached the same level of methodological standardization or bioanalytical sensitivity as more recent evaluations and including them likely increased the variability of our observations.

In conclusion, this review of index substrate DDI evaluations showed significant variability in the percentages and types of studies that include metabolite measurements depending on the substrate drugs considered and highlighted the need for careful consideration when designing such a study and when interpreting PK results.

## Figures and Tables

**Table 1:** Number of index DDI studies with metabolite measurement.

Enzyme	Index Substrate	Index DDI Study	Metabolite Measured (%)	Cocktail Study	Metabolite Measured (%)
CYP1A2	caffeine	474	300 (63%)	195	132 (68%)
CYP2B6	bupropion	112	92 (82%)	15	15 (100%)
CYP2B6	(R)-bupropion	13	12 (92%)	8	8 (100%)
CYP2B6	(S)-bupropion	15	14 (93%)	8	8 (100%)
CYP2C8	repaglinide	132	38 (29%)	6	0
CYP2C9	flurbiprofen ^1^	47	40 (85%)	20	15 (75%)
CYP2C9	(S)-warfarin	390	41 (11%)	55	10 (18%)
CYP2C19	omeprazole	331	201 (61%)	143	95 (66%)
CYP2C19	(R)-omeprazole	2	2 (100%)	0	0
CYP2C19	esomeprazole ^2^	20	4 (20%)	0	0
CYP2D6	dextromethorphan	263	215 (82%)	106	78 (74%)
CYP2D6	desipramine	81	43 (53%)	0	0
CYP2D6	nebivolol	11	7 (63%)	0	0
CYP2D6	(d)-nebivolol	13	0	0	0
CYP2D6	(l)-nebivolol	13	0	0	0
CYP3A	midazolam	1283	468 (36%)	241	132 (55%)
CYP3A	triazolam	61	5 (8%)	0	0
	all	3261	1466 (45%)	797	477 (60%)

^1^ Both (R)- and (S)-enantiomers were also searched in the DIDB but no DDI studies measured them. ^2^ Esomeprazole is the S-isomer of omeprazole

**Table 2:** Number of drugs including clinical index studies and metabolite measurement approved by the FDA from 2014 to 2023.

Enzyme	Index Substrate	Clinical Index Study	Metabolite Measured (%)
CYP1A2	caffeine	16	5 (31%)
CYP2B6	bupropion	10	3 (30%)
CYP2C8	repaglinide	11	0
CYP2C9	(S)-warfarin	24	0
CYP2C9	flurbiprofen	3	1 (33%)
CYP2C19	omeprazole	20	4 (20%)
CYP2D6	desipramine	2	0
CYP2D6	dextromethorphan	11	4 (36%)
CYP2D6	nebivolol	0	0
CYP3A	midazolam	100	35 (35%)
CYP3A	triazolam	0	0

## Data Availability

Data is contained within the article or Supplementary Material.

## Supplementary Materials

The following supporting information can be downloaded at: https://www.mdpi.com/article/10.3390/metabo14100522/s1, Figure S1: Number of studies with metabolite PK data per year from literature and NDAs; Figure S2: Correlation between midazolam AUC fold-change and 1′-hydroxymidazolam/midazolam AUC ratio fold-change in CYP3A inhibition studies; Figure S3: Correlation between midazolam AUC fold-change and 1′-hydroxymidazolam AUC fold-change in CYP3A inhibition studies.
