# Supplementary material for: Metabolite Measurement in Index Substrate Drug Interaction Studies: A Review of the Literature and Recent New Drug Application Reviews
Source: Metabolites. 2024 Sep 26;14(10):522. doi: 10.3390/metabo14100522 (PMC11509402; doi:10.3390/metabo14100522)
Supplement: Supplementary file 1 [file metabolites-14-00522-s001.zip › metabolites-3169744-supplementary.pdf]

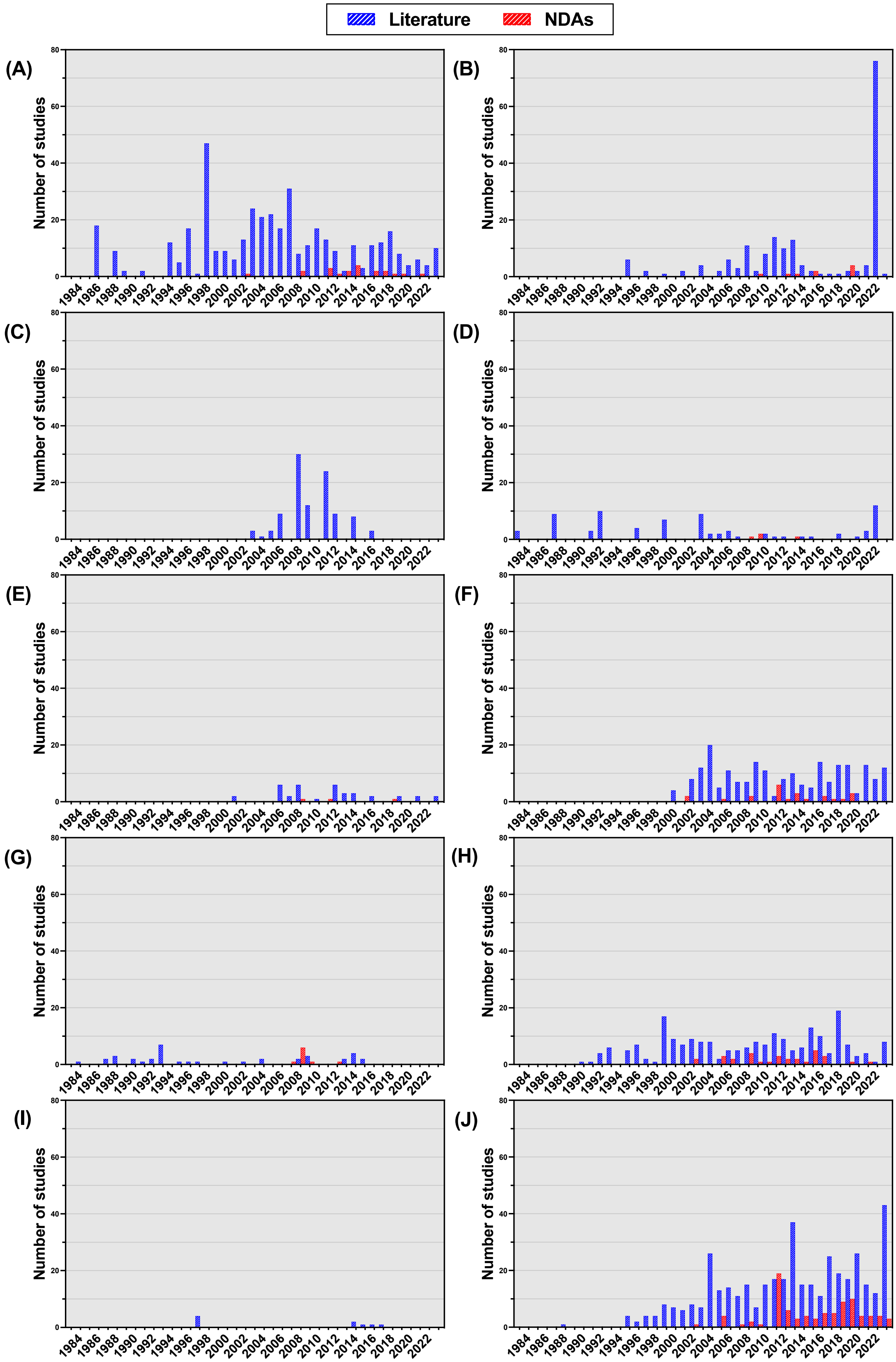

Figure S1. Number of studies with metabolite PK data per year from literature and NDAs for: (A) caffeine, (B) bupropion, (C) repaglinide, (D) (S)-warfarin, (E) flurbiprofen, (F) omeprazole, (G) desipramine, (H) dextromethorphan, (I) nebivolol, and (J) midazolam.

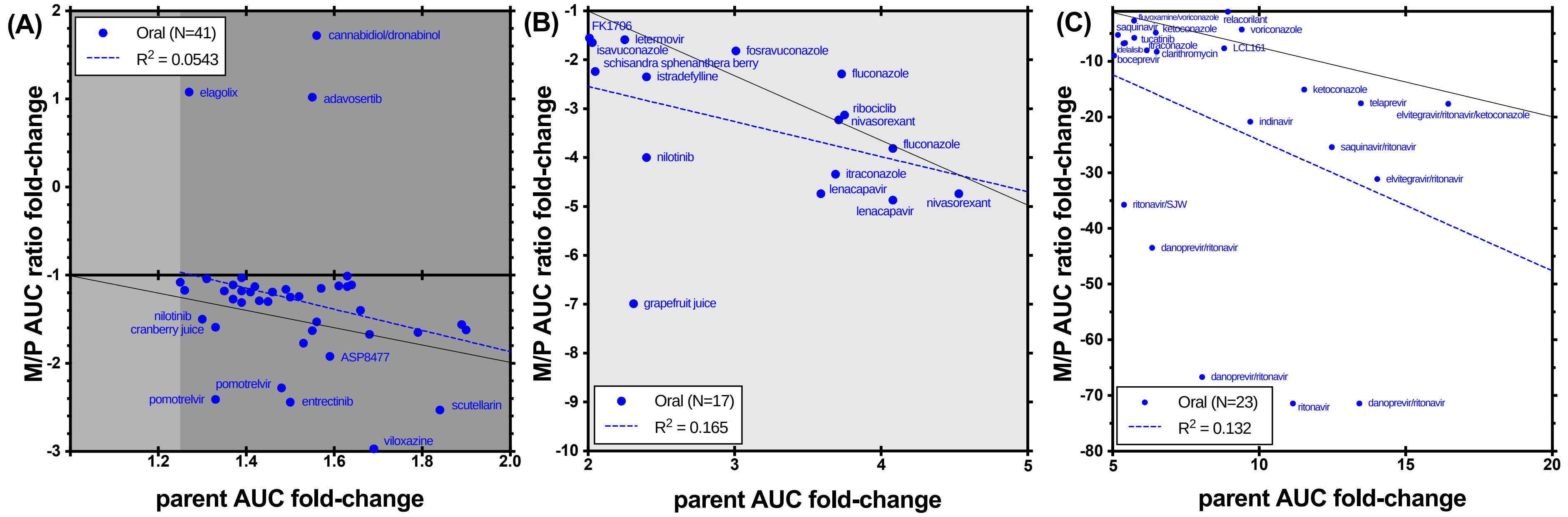

Figure S2. Correlation between midazolam AUC fold-change and 1′-hydroxymidazolam/midazolam AUC ratio fold-change in CYP3A inhibition studies. The black line represents unity, dashed blue lines represent simple linear regression analysis. **(A)** weak inhibition studies with parent AUC fold-change ≥ 1.25- to < 2.0-fold; **(B)** moderate inhibition studies with parent AUC fold-change ≥ 2.0- to < 5.0-fold; **(C)** strong inhibition studies with parent AUC fold-change ≥ 5.0-fold.

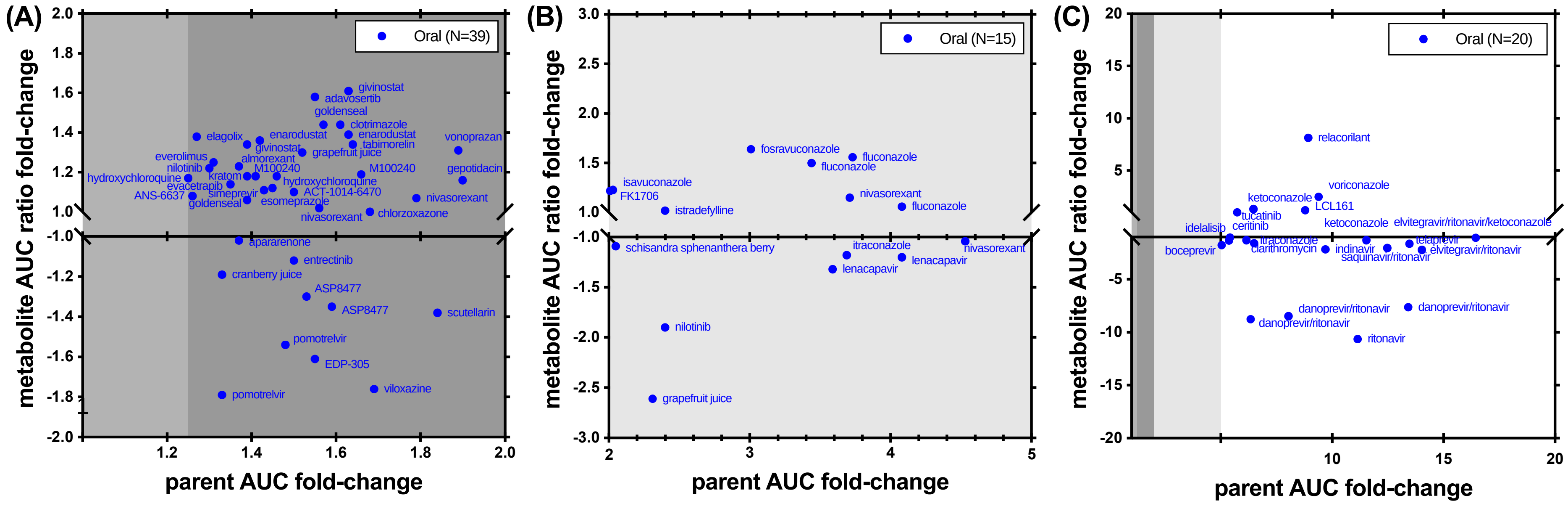

Figure S3. Correlation between midazolam AUC fold-change and 1′-hydroxymidazolam AUC fold-change in CYP3A inhibition studies. The black line represents unity, dashed blue lines represent simple linear regression analysis. **(A)** weak inhibition studies with parent AUC fold-change ≥ 1.25- to < 2.0-fold; **(B)** moderate inhibition studies with parent AUC fold-change ≥ 2.0- to < 5.0-fold; **(C)** strong inhibition studies with parent AUC fold-change ≥ 5.0-fold.
